# Adenylyl Cyclases as Therapeutic Targets in Neuroregeneration

**DOI:** 10.3390/ijms26136081

**Published:** 2025-06-25

**Authors:** Julia Tomczak, Agnieszka Kapsa, Tomasz Boczek

**Affiliations:** Department of Molecular Neurochemistry, Faculty of Health Sciences, Medical University of Lodz, 92215 Lodz, Poland; julia.tomczak1@stud.umed.lodz.pl (J.T.); agnieszka.kapsa@umed.lodz.pl (A.K.)

**Keywords:** adenylyl cyclase (AC), cyclic adenosine monophosphate (cAMP) signaling, neuroregeneration

## Abstract

Adenylyl cyclases (ACs) are key regulators of cyclic adenosine monophosphate (cAMP) signaling—a pathway critical for neuroregeneration, synaptic plasticity, and neuronal survival. In both the central and peripheral nervous systems, injury-induced activation of ACs promotes axonal outgrowth and functional recovery through the stimulation of protein kinase A (PKA), exchange proteins directly activated by cAMP (Epac), and cAMP-response element-binding protein (CREB). Among the various AC isoforms, calcium-sensitive AC1, AC8, and AC5, as well as bicarbonate-responsive soluble AC (sAC), have emerged as crucial mediators of neuroplasticity and axon regeneration. These isoforms coordinate diverse cellular responses—including gene transcription, cytoskeletal remodeling, and neurotransmitter release—to metabolic, synaptic, and injury-related signals. Dysregulation of AC activity has been implicated in the pathophysiology of neurodegenerative diseases such as Parkinson’s disease, Alzheimer’s disease, and amyotrophic lateral sclerosis, as well as in chronic pain syndromes. Pharmacological modulation of cAMP levels through AC activation, phosphodiesterase (PDE) inhibition, or pituitary adenylyl cyclase-activating polypeptide (PACAP) receptor signaling has shown therapeutic promise in preclinical models by enhancing neurogenesis, remyelination, and synaptic repair. Conversely, targeted inhibition of specific AC isoforms, particularly AC1, has demonstrated efficacy in reducing maladaptive plasticity and neuropathic pain. This review highlights the diverse roles of ACs in neuronal function and injury response and discusses emerging strategies for their therapeutic targeting.

## 1. Introduction to Cyclic Adenosine Monophosphate (cAMP)-Dependent Signaling in Nerve Regeneration

Nerve regeneration is a critical biological process with profound implications for the recovery of function following injury to the nervous system. While the peripheral nervous system (PNS) exhibits a notable capacity for self-repair, regeneration in the central nervous system (CNS) is markedly limited. This difference is a result of both intrinsic neuronal factors and the extrinsic inhibitory environment in the CNS. Understanding these mechanisms is essential for developing therapeutic strategies aimed at promoting neuroregeneration, especially in cases of traumatic brain injury, spinal cord injury, or neurodegenerative diseases. At the cellular level, the failure of CNS neurons to regenerate is due in part to the presence of inhibitory molecules such as Nogo-A, myelin-associated glycoprotein (MAG), and oligodendrocyte-myelin glycoprotein (OMgp), as well as the formation of a glial scar rich in chondroitin sulfate proteoglycans (CSPGs), which collectively hinder axonal extension [1]. However, the inability of mature CNS neurons to activate an intrinsic growth program also plays a central role. In contrast, peripheral neurons respond to injury with the upregulation of regeneration-associated genes and activation of signaling pathways that support axonal outgrowth, including the 3′,5′-cyclic adenosine monophosphate (cAMP) signaling cascade. The cAMP pathway is one of the most extensively studied intracellular signaling pathways involved in axon regeneration. It plays a key role in translating injury signals into a regenerative response. Upon neuronal injury, elevated cAMP levels are observed in peripheral neurons, where they correlate with enhanced axonal elongation, branching, and target reinnervation. This regenerative effect of cAMP is largely mediated through activation of the protein kinase A (PKA), which phosphorylates transcription factors such as the cAMP- response element-binding protein (CREB), leading to transcription of regeneration-associated genes [2,3]. In experimental models, artificially elevating cAMP levels in CNS neurons has been shown to partially overcome their regenerative failure. This is achieved either by direct app cation of cAMP analogs like dibutyryl-cAMP or through pharmacological inhibition of phosphodiesterases (PDEs), which break down cAMP. For instance, administration of rolipram, a phosphodiesterase 4 (PDE4) inhibitor, results in sustained cAMP signaling and has been associated with improved axon regrowth and functional recovery following spinal cord injury [4]. Moreover, cAMP acts synergistically with neurotrophic factors such as the brain-derived neurotrophic factor (BDNF) and nerve growth factor (NGF), enhancing their effects on axon extension and survival. cAMP signaling also influences cytoskeletal remodeling, growth cone dynamics, and the expression of integrins—key components in axonal guidance and substrate interaction. Beyond PKA, exchange proteins are directly activated by cAMP (Epac), which modulate small GTPases like Rap1 and Rac1, further contributing to axon elongation and directional growth. This multifaceted downstream signaling allows cAMP to orchestrate a coordinated regenerative response that integrates metabolic, structural, and gene expression changes [5,6].

**Figure 1:**
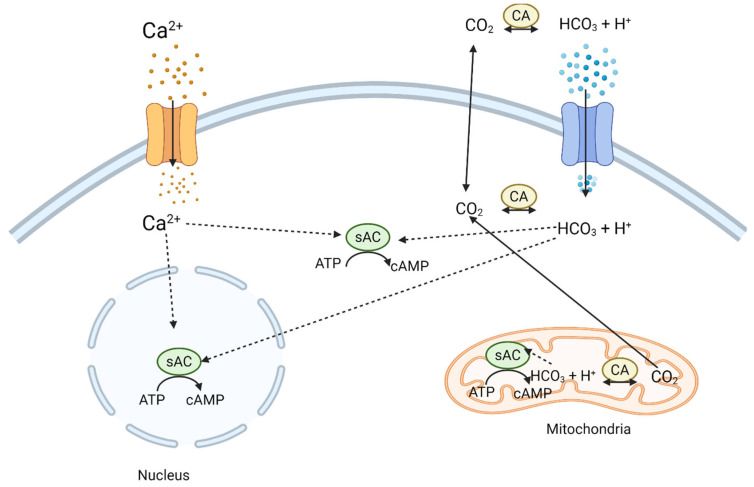
Diagram illustrating the key regulatory and activation mechanisms of soluble adenylyl cyclase (sAC) in a eukaryotic cell. sAC, located in the cytosol, nucleus, and mitochondrial matrix, is activated by calcium ions (Ca^2+^) and bicarbonate (HCO_3_^−^). The influx of Ca^2+^ stimulates sAC in the cytosol and nucleus to produce cAMP from ATP. Bicarbonate, generated from carbon dioxide (CO_2_) by carbonic anhydrase (CA) or transported into the cell, activates sAC in the cytosol and mitochondria. In the nucleus, sAC-dependent cAMP can regulate transcription, while in the mitochondria, it influences metabolism and mitochondrial dynamics. Created in BioRender. Tomczak, J. (2025) https://BioRender.com/mt2mtgo.

Importantly, the source and regulation of cAMP within neurons are tightly controlled by adenylyl cyclases (ACs)—enzymes that catalyze the conversion of ATP into cAMP. In mammals, nine membrane-bound AC isoforms (AC1–AC9) and one soluble isoform (sAC or AC10) are known. Each isoform displays distinct regulatory properties and tissue distribution, and several are prominently expressed in the nervous system (Table 1). Notably, calcium/calmodulin-sensitive isoforms, such as AC1 and AC8, are activated in response to neuronal activity and play an important role in synaptic plasticity and memory formation. Their involvement in activity-dependent cAMP production makes them attractive targets for enhancing neuroregeneration in an injury-responsive manner [7,8]. The soluble adenylyl cyclase, which is activated by bicarbonate and intracellular pH changes, has emerged as a potential metabolic sensor within neurons (Figure 1). Its activity links cellular energy state to cAMP production and downstream signaling, providing a mechanism by which metabolic stress or injury-induced changes in intracellular conditions can influence regeneration. This adds another layer of complexity and potential for therapeutic targeting, as modulation of sAC could potentially enhance regeneration by synchronizing energy metabolism with growth-promoting signaling cascades. Further evidence for the role of cAMP in regeneration comes from studies using conditioning lesion models, where a prior injury to the peripheral branch of a dorsal root ganglion (DRG) neuron enhances the ability of its central branch to regenerate within the spinal cord [9,10]. This phenomenon is associated with a sustained increase in cAMP levels and upregulation of regeneration-associated genes, underscoring the central role of the cAMP pathway in initiating and maintaining a growth-competent state. Despite these advances, successful translation of cAMP-based therapies into clinical practice remains challenging due to several issues such as transient effects, lack of isoform specificity, and potential side effects of systemic cAMP elevation. Thus, future research should be increasingly focused on targeting specific AC isoforms that are enriched in neurons and activated under regenerative conditions. Understanding how each AC isoform contributes to regeneration, both under normal and pathological conditions, will be the key to designing precise and effective treatments [7,11].

## 2. Adenylyl Cyclase in Neuroplasticity

Neuroplasticity, the brain’s ability to modify its structure and function in response to experience, injury, or disease, is a fundamental property that underlies learning, memory, sensory adaptation, and recovery from neural damage [24]. This process occurs at multiple levels, ranging from molecular and synaptic changes to large-scale cortical reorganization [25]. One of the key mechanisms of neuroplasticity is synaptic plasticity, which refers to the ability of synapses to strengthen or weaken over time in response to activity. Synaptic plasticity is broadly classified into short-term and long-term plasticity. Short-term plasticity involves transient modifications in synaptic strength, typically lasting milliseconds to minutes, and is primarily mediated by changes in presynaptic neurotransmitter release. In contrast, long-term plasticity, such as long-term potentiation (LTP) and long-term depression (LTD), leads to persistent modifications in synaptic efficacy, and is thought to be the cellular basis of learning, memory, and experience-dependent adaptations [26]. LTP is defined as a long-lasting increase in synaptic strength following high-frequency stimulation of presynaptic neurons. This phenomenon has been extensively studied in key brain regions such as the hippocampus, anterior cingulate cortex (ACC), and the amygdala [27]. The induction of LTP requires calcium influx, which occurs primarily through N-metyl-D-aspartate (NMDA) receptors and, in some cases, through L-type voltage-gated calcium channels (L-VDCCs) [28]. The elevation in intracellular calcium triggers a cascade of molecular events that lead to synaptic strengthening, including activation of calcium/calmodulin (CaM)-dependent signaling pathways, recruitment of alpha-amino-3-hydroxy-5-methyl-4-isoxazolepropionic acid receptors (AMPA) to the synaptic membrane, and structural modifications of dendritic spines [29].

**Figure 2:**
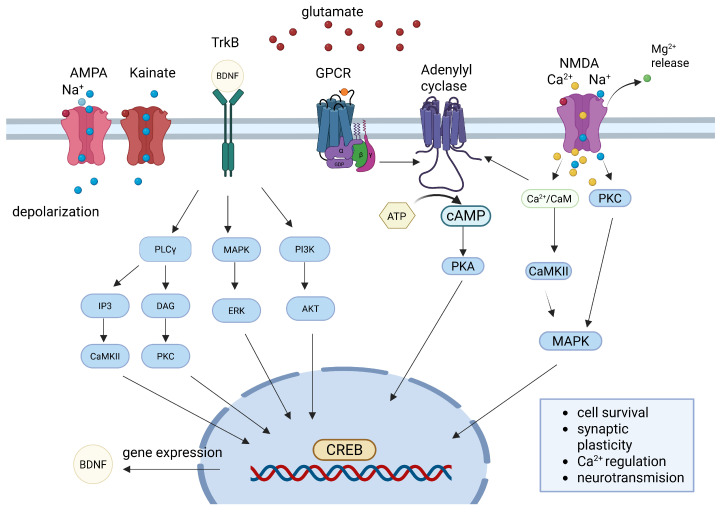
cyclic adenosine monophosphate (cAMP)/protein kinase A (PKA)/cAMP-response element-binding protein (CREB) and brain-derived neurotrophic factor (BDNF) signaling pathways involved in synaptic plasticity. Synaptic activity triggered by glutamate release activates ionotropic receptors-AMPA (α-amino-3-hydroxy-5-methyl-4-isoxazolepropionic acid), NMDA (N-methyl-D-aspartate), and kainate-as well as GPCRs (G-protein-coupled receptors), leading to membrane depolarization and calcium ion (Ca^2+^) influx. GPCR stimulation enhances cAMP production via adenylyl cyclase (AC), activating PKA, which phosphorylates CREB. In parallel, BDNF binding to tropomyosin receptor kinase B (TrkB) receptors activates mitogen-activated protein kinase (MAPK)/extracellular signal-regulated kinase (ERK), phosphoinositide 3-kinases (PI3K)/AKT, and phospholipase C gamma (PLCγ) pathways, which also converge on CREB. Activated CREB drives transcription of genes essential for synaptic plasticity, including BDNF itself. These signaling cascades regulate key processes such as synapse strengthening, calcium homeostasis, neurotransmission and neuronal survival. Created in BioRender. Tomczak, J. (2025) https://BioRender.com/mt2mtgo.

At the molecular level, long-term synaptic plasticity is governed by complex intracellular signaling pathways that translate synaptic activity into long-lasting functional and structural changes. One of the key signaling molecules in this process is cAMP, a second messenger that plays a crucial role in activity-dependent neuronal modifications (Figure 2). cAMP and its downstream effector, CREB, play pivotal roles in synaptic plasticity, particularly in the formation and maintenance of LTP, a fundamental mechanism underlying learning and memory. Upon synaptic stimulation, elevated cAMP levels activate PKA, which phosphorylates CREB at serine 133. This modification enables CREB to bind cAMP response elements (CRE) in the promoters of target genes, initiating transcription. The expression of these genes is essential for producing proteins involved in synapse formation, dendritic spine remodeling, and long-term memory consolidation [30]. Experimental models support the central role of CREB in neuroplasticity. Mice expressing a dominant-negative form of CREB exhibit deficits in LTP and long-term memory, while those overexpressing active CREB show enhanced synaptic strengthening and improved memory performance [31,32]. In addition to promoting gene expression necessary for synaptic growth, CREB also modulates neuronal excitability and integrates diverse signaling inputs over time, thereby contributing to both the encoding and retrieval of memory traces [33,34]. Furthermore, CREB activity facilitates the expression of BDNF, a crucial modulator of synaptic strength and neuronal survival [35,36].

In the hippocampus, NMDA receptor activation leads to calcium influx, which stimulates calcium/calmodulin-dependent AC activity, thereby promoting cAMP production. This signaling pathway is crucial for the induction and maintenance of LTP, as it facilitates the molecular changes required for long-term synaptic modifications [7,37]. The cAMP cascade also interacts with voltage-gated calcium channels, creating a feedback loop that further amplifies the calcium influx and enhances synaptic potentiation. These mechanisms are observed in multiple hippocampal pathways, including the Schaffer collateral, mossy fiber, and medial perforant pathways, where AC1 and AC8 cooperate to facilitate memory consolidation [38,39].

Adenylyl cyclase also plays a pivotal role in cortical plasticity, particularly in the ACC, where synaptic modifications contribute to sensory processing, learning, and pain perception [40,41]. Both AC1 and AC8 contribute to synaptic potentiation in the ACC, but AC1 plays a more prominent role in NMDA receptor-dependent LTP [42]. Given its high sensitivity to calcium, AC1 serves as a major calcium sensor for the NMDA receptor and L-VDCC-mediated signaling in ACC neurons. The resulting cAMP production triggers downstream pathways that support LTP induction and maintenance, reinforcing the role of AC1 as a central regulator of synaptic plasticity [41,43,44]. AC1 is essential for cortical development and sensory processing. Studies using AC1 knockout models have revealed significant deficits in the functional maturation of thalamocortical synapses and alterations in the barrel cortex cytoarchitecture, suggesting that AC1 is necessary for proper sensory circuit formation [45,46]. Furthermore, AC1 has been implicated in experience-dependent plasticity in the thalamus, where it contributes to the strengthening of whisker relay synapses in response to sensory input [47].

Beyond its role in normal plasticity, adenylyl cyclase-mediated signaling is critically involved in injury-induced neuroplasticity, particularly in conditions such as chronic pain. Following nerve injury, synaptic connections in the ACC undergo maladaptive changes that contribute to the persistence of pain. These injury-induced modifications involve both presynaptic and postsynaptic alterations, including enhanced glutamate release, increased AMPA receptor-mediated responses, and upregulated phosphorylation of glutamate receptor 1 (GluR1) subunits [48,49,50]. AC1 has been identified as a crucial regulator of synaptic plasticity in chronic pain conditions, as its activity is required for the induction of LTP in various brain regions [40,42,51]. In the ACC, a key region involved in pain perception, AC1 mediates both presynaptic and postsynaptic plasticity following peripheral nerve injury. Increased AC1 activity leads to enhanced presynaptic glutamate release and upregulation of postsynaptic AMPA receptor responses in layer II/III neurons of the ACC, contributing to long-term pain sensitization [48]. Studies have demonstrated that AC1 knockout mice show significant reductions in neuropathic pain-related synaptic potentiation, further supporting the role of AC1 in amplifying pain-related plasticity in the ACC [52,53]. The ability of AC1 to couple calcium signaling to cAMP production makes it a central player in both forms of LTP, driving synaptic modifications that underlie chronic pain [51,54]. Pharmacological inhibition of AC1 has been shown to prevent pain-related synaptic potentiation, block behavioral sensitization, and reduce injury-induced anxiety [54]. Unlike broad-spectrum cAMP modulators, AC1 inhibitors selectively disrupt maladaptive plasticity while preserving normal synaptic function, making them attractive candidates for therapeutic interventions in chronic pain and related conditions [54,55]. The selective AC1 inhibitor NB001 has been shown to effectively block LTP induction in the ACC and insular cortex without affecting thee basal synaptic transmission, demonstrating its potential for selectively targeting maladaptive plasticity while preserving normal synaptic function [51,56]. While AC1 is the dominant player in injury-induced plasticity, AC8 also contributes to synaptic modifications, although its function appears to be more specialized. Unlike AC1, which is highly sensitive to calcium influx and required for NMDA receptor-dependent LTP, AC8 is involved in forskolin-induced potentiation and synaptic depotentiation [40,41,42]. In neuropathic pain models, AC8 mRNA expression increases in the ACC, suggesting that it may have a role in maintaining injury-induced plasticity [40].

## 3. Adenylyl Cyclase Signaling in Axonal Regrowth and Nerve Repair

Adenylyl cyclases play a central role in the regulation of axonal outgrowth by generating cAMP involved in numerous cellular processes including neurite extension, growth cone dynamics, and axon regeneration. Among the AC family, soluble adenylyl cyclase has gained attention as a critical mediator of axonal growth [10]. sAC has been shown to play a pivotal role in retinal ganglion cell (RGC) survival and axon regeneration. Its activation, either through physiological stimuli, such as electrical activity or direct overexpression, significantly enhances axon growth and neuronal survival both in vitro and in vivo. In contrast, the inhibition of sAC dramatically reduces these regenerative outcomes, underscoring its essential role. Notably, transmembrane AC isoforms AC1 and AC8 are not required for these effects, suggesting that sAC is the predominant source of cAMP involved in these regenerative pathways [57,58]. sAC-generated cAMP is particularly effective at promoting neurite outgrowth on inhibitory substrates, such as CNS myelin [9]. For example, sAC is necessary for the BDNF-mediated overcoming of MAG-induced inhibition, and elevating sAC levels in neurons sufficiently promotes axonal growth on myelin in vitro and regeneration in vivo. This highlights sAC as a key intracellular transducer capable of converting extracellular growth cues into pro-regenerative signals through cAMP production [59,60]. Netrin-1 signaling also intersects with sAC-mediated pathways during axonal development and regenerative axon growth. Netrin-1 is a bifunctional guidance cue that can attract or repel axons during neural development, depending on the receptor context [61,62,63]. This bifunctionality is mediated by two receptor families: DCC (Deleted in Colorectal Cancer) and UNC-5 homologues [64,65]. Netrin-1 is implicated in various processes, including axon branching, guidance, and peripheral nerve regeneration. In developing cortical neurons, netrin-1 dramatically increases axon branching through calcium transients that activate the downstream effectors calcium/calmodulin-dependent protein kinase II (CaMKII) and mitogen-activated protein kinase (MAPK) [66]. Wu et al. (2006) [67] showed that sAC is expressed in developing rat axons and produces cAMP in response to netrin-1. Overexpression of sAC promoted axonal outgrowth and growth cone elaboration, while its inhibition suppressed netrin-1-induced responses, indicating that sAC may mediate key aspects of netrin-1-dependent axon development [67]. However, this role remains debated. Moore et al. (2008) reported only weak sAC expression in embryonic neurons and observed normal axonal pathfinding in sAC-null mice, suggesting that sAC is not essential for all netrin-1-mediated guidance functions [61]. These conflicting findings suggest that the contribution of sAC to netrin-1 signaling may be context- or cell-type-dependent or that compensatory mechanisms exist. Beyond sAC, specific transmembrane adenylyl cyclase isoforms also play a crucial role in regulating axonal growth and neuronal function. AC6 plays an inhibitory role in neurite extension through its interaction with the Snapin–SNAP-25 complex, which is essential for neurosecretion and neurite extension. Synaptosomal-associated protein-25 (SNAP-25), a critical component of the SNARE complex, facilitates synaptic vesicle exocytosis and supports neurite outgrowth [68,69,70,71]. SNAP-associated protein (Snapin) binds to SNAP-25, enhancing its interaction with synaptotagmin, which stabilizes release-ready vesicles and modulates the process of neurosecretion. The presence of Snapin is vital for calcium-dependent exocytosis, and its absence significantly reduces exocytotic activity in cells, such as chromaffin cells [72]. The overexpression of AC6 suppresses neurite outgrowth, while its knockdown or disruption of its interaction with Snapin promotes neurite extension. This suggests that AC6 regulates neurite growth by modulating the Snapin–SNAP-25 complex, possibly through cAMP signaling pathways that impact the dynamics of synaptic vesicle release and neurite development [73]. The expression of SNAP-25 is regulated by transcription factors, such as the Brn-3a transcription factor, which is critical for proper neurite outgrowth [74]. Additionally, SNAP-25 expression is developmentally regulated in neurons, with higher levels observed in immature neurons and a reduction in adult hippocampal inhibitory synapses [75,76]. Different isoforms of SNAP-25, including SNAP-25a, SNAP-25b, and SNAP-23, contribute to various neuronal functions, including cell survival, dendritic arborization, and neurotransmitter release [69,77]. AC5 plays a pivotal role in coordinating purinergic signaling during axonal elongation. It acts as a key integrator of the effects exerted by different purinergic receptors, notably the P2Y1, P2Y13, and P2X7 receptors. These receptors have contrasting roles in axonal growth, with P2Y1 promoting elongation and P2Y13/P2X7 inhibiting it. Recent studies indicate that AC5 plays a crucial role in balancing the effects of these receptors, primarily by modulating downstream signaling pathways that influence axonal growth. When the P2X7 receptor is inhibited, axonal elongation is enhanced through the activation of several key signaling pathways, including CaMKII, focal adhesion kinase (FAK), and phosphoinositide 3-kinases (PI3K), which contribute to cytoskeletal reorganization and the promotion of axon branching and extension [78,79]. The ability of AC5 to integrate these signals underscores its role as a key modulator of purinergic control over axonal growth. By coordinating the actions of growth-promoting (P2Y1) and growth-inhibiting (P2Y13 and P2X7) receptors, AC5 ensures the fine-tuned regulation of axonal elongation [78,80].

**Figure 3:**
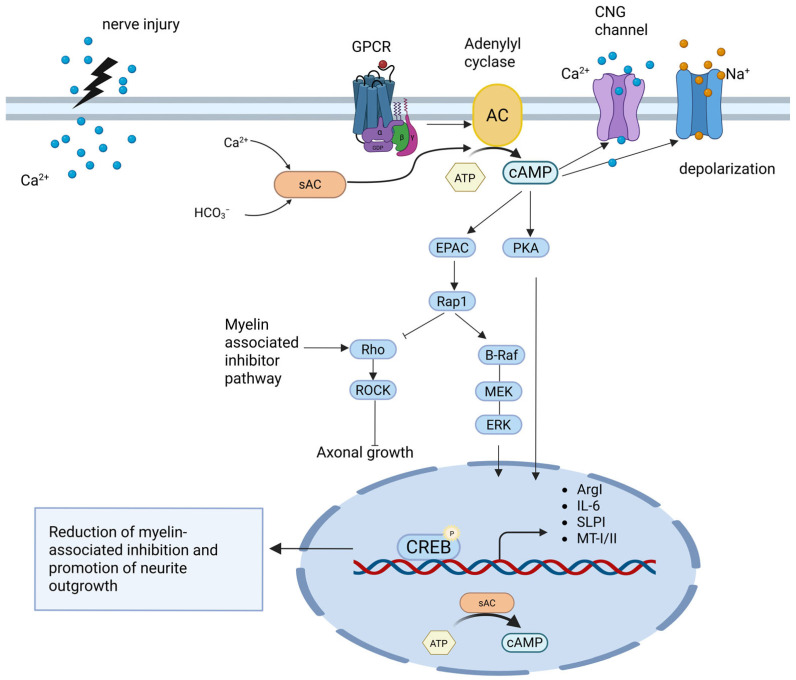
cAMP-mediated signaling pathways promoting axonal regeneration after nerve injury. Nerve injury leads to calcium influx and activation of G-protein-coupled receptors (GPCRs), which stimulate transmembrane adenylyl cyclases (ACs) and soluble AC (sAC) to produce cAMP. Elevated cAMP levels activate two major effectors: protein kinase A (PKA) and exchange protein directly activated by cAMP (Epac). PKA phosphorylates the transcription factor cAMP-response element-binding protein (CREB), promoting the expression of regeneration-associated genes, including arginase I (Arg1), interleukin-6 (IL-6), secretory leukocyte protease inhibitor (SLPI), and metallothionein I/II (MT-I/II). These genes help overcome myelin-associated growth inhibition. In parallel, Epac activates Rap1, which promotes axonal growth through inhibition of the RhoA/Rho-associated kinase (ROCK) pathway and the activation of the B-Raf/MEK/ERK (MAPK) cascade. cAMP signaling also enhances calcium entry via cyclic nucleotide-gated (CNG) channels, contributing to membrane depolarization and further amplifying regenerative responses. Collectively, these pathways reduce extrinsic inhibitory signals and support intrinsic neurite outgrowth and functional recovery. Created in BioRender. Tomczak, J. (2025) https://BioRender.com/mt2mtgo.

In the context of nerve injury, ACs contribute significantly to regeneration in both central and peripheral systems. cAMP is essential for self-recovery, axon regeneration, and remyelination repair [81]. The effects of cAMP are transcription-dependent, mediated through PKA and CREB activation, leading to the upregulation of pro-regenerative genes. These genes include arginase I, interleukin-6, the secretory leukocyte protease inhibitor, and metallothionein-I/II, which have shown potential in overcoming myelin-mediated inhibition [82]. The activation of ACs at injury sites and in regenerating nerve tips suggests their involvement in conditioning the local environment for effective nerve repair [2,83] (Figure 3). In the central nervous system, ACs play a crucial role in spinal cord injury (SCI) recovery by regulating neuroplasticity, axonal regeneration, and pain mechanisms. Among the various AC isoforms, calcium-sensitive AC1 and AC8 have been identified as key regulators of both SCI recovery and neuropathic pain. AC1, in particular, is critical for corticospinal tract development and repair, as its deficiency alters corticospinal motor neuron density and enhances locomotor recovery following SCI. This suggests that AC1-mediated cAMP signaling may influence motor circuit reorganization and functional recovery [84]. AC1 also contributes to neuronal signaling in the spinal dorsal horn, where it facilitates extracellular signal-regulated kinase (ERK) activation—a pathway implicated in spinal sensitization and the persistence of pain following SCI [85]. Additionally, AC1 and AC8 regulate cAMP-dependent signaling in the ACC, a region involved in pain perception and emotional responses to injury. Interestingly, while AC1 expression remains unchanged after nerve injury, AC8 mRNA is significantly upregulated in NMDA receptor 2B-positive neurons in the contralateral ACC, suggesting a potential role in synaptic remodeling and the development of neuropathic pain [40]. AC activation has been shown to promote neuroprotection and regeneration following SCI. Pharmacological approaches that elevate intracellular cAMP, such as the administration of meglumine cyclic adenylate, have been demonstrated to improve neurological function by activating AC3 and suppressing phosphodiesterase 4D (PDE4D), thereby enhancing axonal regrowth and functional recovery [86,87]. Additionally, spinal cord transection results in increased dopamine-activated AC sensitivity, implicating ACs in normal spinal function and potential neuroleptic drug side effects [88]. Recent research also suggests that targeting the cAMP effector exchange protein directly activated by cAMP 2 (Epac2) could be a novel strategy for promoting spinal cord repair, as Epac2 influences cytoskeletal dynamics and cellular adhesion—processes critical for neuronal regeneration after injury [89]. These findings further support the potential therapeutic applications of AC modulation in neural repair. Following SCI, persistent spontaneous activity in primary nociceptors is maintained by scaffolded AC and PKA complexes, particularly AC5 and AC6, which interact with the A-kinase anchoring protein 150 (AKAP150). This persistent excitability is believed to contribute to central sensitization, a mechanism underlying chronic pain. Moreover, SCI leads to increased AKAP150 expression and altered AC regulation, with enhanced calcium–calmodulin stimulation and reduced Gαi inhibition of AC activity, further amplifying pain signaling pathways [90]. Recent findings indicate that the perinuclear scaffold mAKAP (also known as AKAP6) is more closely linked to neuroprotection and axon growth. This perinuclear cAMP compartment is regulated by local Ca^2+^ signaling, which mediates activity-dependent cAMP elevation and subsequent PKA activation [91]. Importantly, genetic deletion of mAKAPα or disruption of its associated cAMP signaling impairs the neuroprotective effects of neurotrophic factors and cAMP elevation, while enhancing mAKAPα-associated cAMP signaling increases RGC survival and axon growth, both in vitro and in vivo [4,92]. More recently, mAKAPα was shown to regulate calcineurin/MEF2 signaling to drive activity-dependent axon elongation in hippocampal neurons [93].

Recent studies have highlighted the crucial role of ACs in optic nerve regeneration. sAC serves as a primary source of cAMP, allowing BDNF to overcome MAG-mediated inhibition of neurite outgrowth. In vitro studies demonstrate that directly elevating sAC levels in neurons is sufficient to stimulate neurite outgrowth on myelin substrates, while in vivo experiments show that sAC activation promotes axonal regeneration [60]. Furthermore, sAC is responsive to bicarbonate and cations, suggesting that it acts as a metabolic and electrical sensor, integrating physiological signals to determine whether a neuron survives or regenerates following injury [10].

Recent research has identified the AC8 family member Ac78C as a crucial regulator of cAMP production following dendritic injury in Drosophila neurons. Ac78C acts downstream of voltage-gated calcium channels to promote timely initiation of dendrite regeneration. Calcium and cAMP accumulate in the cell body after both dendrite and axon injury. Two voltage-gated calcium channels (L-Type and T-Type) are required for the calcium influx in response to dendrite injury and play a role in the rapid initiation of dendrite regeneration. The adenylyl cyclase Ac78C is required for cAMP production after dendrite injury and timely initiation of regeneration, and this cAMP production is dependent on the calcium influx mediated by the VDCCs [94]. This finding aligns with previous studies on mammalian AC8, which is known to be stimulated by calcium-bound calmodulin [95]. Activation of the cAMP pathway activates synapses that are silent at rest, while the inhibition of cAMP signaling silences basally active synapses. The Ca^2+^-sensitive adenylyl cyclase isoform AC8, but not AC1, plays a particularly important role in the recovery of synaptic function after strong presynaptic silencing [96]

In peripheral nerve injury, AC activity is dynamically regulated, influencing the regeneration process. Following nerve damage, AC activity increases at the site of injury and in regenerating axons, particularly in conditioned-lesion nerves where an additional injury had previously occurred. This preconditioning effect is associated with elevated cAMP levels in nerve segments distal to the lesion, suggesting a role in enhancing axon elongation and functional recovery [83,97]. In addition to neurons, other cells, such as macrophages, Schwann cells, astrocytes, microglia, and endothelial cells, also exhibit AC activity near the injury site, indicating a broader involvement in the regeneration process [81]. The regenerative capacity of peripheral nerves relies on a cascade of molecular events, including calcium signaling, transcription factor activation, inflammation, and the expression of neurotrophic factors. cAMP is a critical molecular determinant in this process, influencing both neurons and Schwann cells to drive axonal regrowth and reinnervation [98]. However, peripheral nerve injury often leads to reduced cAMP levels due to decreased AC activity and increased phosphodiesterase-mediated cAMP degradation [5,99]. This decline in cAMP may impair regenerative processes, highlighting the importance of therapeutic strategies aimed at restoring AC activity or inhibiting cAMP breakdown to enhance recovery.

## 4. Implications of AC Modulation in Neurodegenerative Diseases

Recent research has increasingly emphasized the critical role of adenylyl cyclases in the pathogenesis and potential treatment of neurodegenerative diseases. In neurodegenerative diseases, such as Parkinson’s disease (PD), Alzheimer’s disease (AD), amyotrophic lateral sclerosis (ALS), spinal muscular atrophy (SMA), and spinobulbar muscular atrophy (SBMA), increasing evidence indicates that alterations in AC activity—whether isoform-specific, spatial, or stimulus-dependent—play a central role in disease progression and may serve as promising therapeutic targets.

In Parkinson’s disease, disruptions in cAMP signaling have been documented at multiple levels. One of the most compelling findings is the dysregulation of soluble adenylyl cyclase in parkin-mutant fibroblasts, where basal cAMP levels are significantly elevated. This increase appears to stem from both enhanced sAC activity and decreased activity of phosphodiesterase 4 (PDE4) [99,100]. Parkin, an E3 ubiquitin ligase, has been shown to regulate mitochondrial calcium homeostasis by promoting the proteasomal degradation of the mitochondrial calcium uptake 1 (MICU1), a regulator of the mitochondrial calcium uniporter [99,101]. In the absence of parkin, increased MICU1 stability results in elevated mitochondrial calcium uptake, which may activate sAC as a compensatory response to mitigate mitochondrial oxidative stress. This triggers a feedback loop where increased cAMP and PKA activity transiently enhances mitochondrial function by phosphorylating the components of the electron transport chain, particularly complexes I and IV. However, chronic activation of this pathway can lead to reactive oxygen species (ROS) overproduction and further oxidative damage [99,102]. In parallel, animal models of PD further underscore the relevance of AC-mediated cAMP signaling. In a Drosophila model exposed to the mitochondrial toxin rotenone, selective activation of Gαs-coupled receptors in dopaminergic neurons—which stimulates AC and raises intracellular cAMP—rescued locomotor deficits and prevented dopaminergic neuron degeneration, whereas the activation of Gαi-coupled receptors—which inhibit AC activity and reduce cAMP—exacerbated neurodegeneration [103]. Moreover, pharmacological inhibition of PDEs, which degrade cAMP, conferred protection to dopaminergic neurons, further emphasizing the therapeutic potential of boosting cAMP signaling in PD [104,105]. At the level of the basal ganglia circuitry, the importance of AC5, a transmembrane AC isoform, becomes particularly evident. AC5 is predominantly expressed in dopaminergic target areas such as the striatum, where it integrates D1 and D2 receptor signaling in medium spiny neurons [106]. Loss of AC5 in knockout mice results in motor deficits resembling parkinsonian symptoms, including bradykinesia and impaired coordination, indicating that other AC isoforms such as AC1 or AC6 cannot fully compensate for AC5 function [107]. Interestingly, the inhibition of AC5 not only affects baseline motor control but also plays a role in modulating L-DOPA-induced dyskinesia (LID). In models of LID, AC5 inhibition reduces PKA activity and downstreams targets such as FosB/ΔFosB, which are implicated in the maladaptive plasticity associated with chronic L-DOPA therapy [107,108]. The therapeutic potential of modulating AC activity is further illustrated by studies targeting A2A adenosine receptors, which co-localize with D2 dopamine receptors in the striatum and couple with Gs proteins. The A2A receptor activation decreases D2 receptor signaling and indirectly stimulates AC activity; antagonists of A2A receptors enhance D2 receptor function, reduce AC5 activation and improve motor symptoms, offering an alternative or complementary approach to dopamine replacement therapies [109,110,111]. Moreover, emerging regulators of AC5, such as protein phosphatase 2 catalytic subunit beta (PPP2CB) and NSF attachment protein alpha (NAPA), suggest a complex network of protein interactions that modulate AC activity and may serve as novel drug targets [106,112].

In Alzheimer’s disease, alterations in AC signaling appear to follow a different but equally detrimental trajectory. Several studies have reported decreased AC activity reduced in Alzheimer’s disease brains—most notably in the hippocampus and cerebellum—and that this reduction is especially pronounced when AC is stimulated via Gs-coupled β-adrenergic receptors, compared with direct activation of the enzyme [113,114,115]. This suggests a disruption in the G-protein–AC coupling rather than in the catalytic function of AC itself. Notably, this impairment is not due to the postmortem delay or agonal factors but reflects disease-specific changes. Immunohistochemical studies have confirmed reduced expression of AC1 and AC2 subtypes in key brain areas, including the hippocampus and neocortex [116]. The resulting decrease in cAMP production may impair PKA and CREB signaling, which are essential for memory formation and synaptic plasticity [117,118]. Interestingly, experimental studies have shown that AC inhibition can stimulate neurogenesis and improve cognitive function in aged mice, providing a paradoxical but potentially valuable therapeutic avenue [119]. This suggests that precise modulation—rather than simple upregulation or downregulation—of AC activity may yield cognitive benefits depending on disease stage and context.

The cAMP/PKA/CREB pathway is also implicated in ALS. Cortical neurons derived from an ALS patient’s iPSCs show reduced CREB activation, leading to impaired dendritic and synaptic health. This impaired activation correlates with dendritic and synaptic dysfunction and has been linked to an imbalance in PKA subunit expression. Importantly, modulation of cAMP levels has been shown to restore CREB activity and improve neuronal health [120]. The pathway is also involved in regulating TAR DNA-binding protein 43 (TDP-43), a protein central to ALS pathology. TDP-43 aggregation and cytoplasmic mislocalization, both of which contribute to neurodegeneration, are regulated by cAMP/PKA signaling [121]. In mouse models of ALS, activation of the cAMP/PKA pathway has been shown to reverse synaptic deficits in spinal motoneurons, enhance their firing activity, and improve functional disease markers [122]. Moreover, a loss of primary cilia in motor neurons—structures marked by the expression of AC3—has been detected in SOD1-G93A mice, suggesting that the AC3 dysfunction and disrupted cAMP signaling may contribute to disease progression [50]. Further supporting this, downregulation of cAMP/PKA/CREB signaling is increasingly recognized as a pathological feature in ALS. Pharmacological stimulation of this pathway using agents such as forskolin and solanesol has demonstrated neuroprotective effects, likely through enhancement of mitochondrial function [123]. Additionally, PACAP, a neuropeptide that activates adenylyl cyclase, has been shown to promote motor neuron survival through PKA-dependent of epidermal growth factor receptor (EGFR) and upregulation of pro-survival genes, including matrix metallopeptidase 2 (MMP-2) [124].

In SMA and SBMA, enhancing cAMP signaling has emerged as a promising protective strategy. In SMA, clinical severity correlates inversely with the abundance of fully spliced survival motor neuron (SMN) protein and with the number of SMN-positive nuclear “gems” [125]. Elevating intracellular cAMP—either by inhibiting phosphodiesterase-4 with rolipram or by directly activating AC with forskolin—restores exon 7 inclusion in SMN2 transcripts and increases SMN protein and gem counts in patient-derived fibroblasts, supporting cAMP up-regulation as a therapeutic avenue [126,127]. A parallel mechanism operates in SBMA, a polyglutamine-expansion disease of the androgen receptor (AR). Activation of the AC/cAMP/PKA cascade reduces phosphorylation-dependent aggregation of the mutant AR, lessens its toxicity, and improves motor-neuron survival in cell and mouse models [127,128].

## 5. Pharmacological Modulation of AC Activity

Pharmacological modulation of adenylyl cyclase activity has emerged as a powerful strategy to enhance neuroregeneration and counteract maladaptive plasticity. Small molecule activators such as forskolin, 8-bromo cAMP, and rolipram elevate intracellular cAMP and thereby stimulate axonal growth, synaptic remodeling, and functional recovery in a variety of injury models [9,82]. Rolipram, by inhibiting phosphodiesterase 4, prolongs cAMP elevation and yields similar improvements in axon regrowth and functional outcome following spinal trauma [87,129,130]. The membrane-permeant cAMP analog 8-bromo-cAMP mimics the effects of NGF, overcoming myelin-associated inhibition to boost neurite extension [131]. The underlying mechanism involves activation of the MAPK/ERK and PI3K/Akt pathways, along with inactivation of the Rho signaling pathway, all of which contribute to cytoskeletal remodeling and enhanced neurite outgrowth [132]. Transmembrane AC activators have demonstrated significant neuroprotective and regenerative effects. Forskolin, a potent activator of transmembrane ACs, increases cAMP levels and has been shown to accelerate axonal elongation, enhance remyelination, and restore neuronal function in various injury models [133]. In the PNS, forskolin enhances peripheral nerve regeneration by reducing the latency of axonal sprouting initiation and improving Schwann cell function [134,135,136]. In CNS disorders, forskolin has demonstrated therapeutic potential in Parkinson’s disease models by restoring behavioral and neurochemical deficits through activation of the AC/cAMP/PKA-CREB pathway and improving mitochondrial function [137,138]. Forskolin has also been shown to promote remyelination, restore mitochondrial enzymes, and reduce neuroinflammation in multiple sclerosis models [133]. In Alzheimer’s disease models, forskolin improves cognitive function, reduces amyloid-β plaque deposition, and modulates inflammatory responses [139]. Additionally, in dementia models, forskolin attenuates memory deficits and exhibits anticholinesterase, antiamyloid, antioxidative, and anti-inflammatory effects [140]. At the cellular level, forskolin reverses amyloid-β-induced inhibition of long-term potentiation by enhancing cAMP signaling [141]. However, forskolin’s effects on tau phosphorylation are complex. While it activates PKA and increases tau phosphorylation at several sites associated with Alzheimer’s disease, this priming makes tau more susceptible to further phosphorylation by glycogen synthase kinase-3 (GSK-3), suggesting both beneficial and potentially detrimental effects in Alzheimer’s pathology [142,143].

Forskolin has also shown therapeutic benefits in Huntington’s disease models, where it reverses behavioral deficits and restores biochemical parameters in a dose-dependent manner [144]. In an inducible cell model of Huntington’s disease, forskolin partially rescues neurite outgrowth and prevents cell death associated with polyglutamine expansions by enhancing CRE-mediated transcription [145]. Furthermore, forskolin restores motor function and prevents midbrain dopamine neuron loss in a rat model of Parkinson’s disease, demonstrating superiority over standard levodopa treatment as a disease-modifying therapeutic alternative [137]. In multiple sclerosis models, forskolin inhibits inflammatory responses, modulates iron homeostasis, and improves motor and cognitive functions by restoring mitochondrial enzymes and neurotransmitter levels [133,146]. Additionally, forskolin has been explored as a treatment for glaucoma, where it reduces intraocular pressure and stimulates neurotrophic factors, protecting retinal ganglion cells from degeneration [147,148]. Clinical trials have demonstrated forskolin’s effectiveness in lowering intraocular pressure in open-angle glaucoma patients, making it a potential alternative to beta-blockers, particularly in patients with asthma [149]. Forskolin’s neuroregenerative effects extend to spinal cord injury models, where its combination with rosiglitazone enhances locomotor recovery and neuronal repair [150]. Additionally, forskolin synergizes with transforming growth factor beta 1 (TGF-β1) to reactivate chronically denervated Schwann cells, improving axonal regeneration in injured peripheral nerves. This combinational therapy significantly increases the number of regenerated axons at the repair site and reduces Schwann cell dysfunction associated with chronic denervation [151,152]. More recently, attention has turned to the unique properties of soluble AC, which—unlike transmembrane isoforms—responds directly to intracellular bicarbonate and calcium [11,153]. Pharmacological activation of sAC enhances retinal ganglion cell survival and optic nerve regeneration, whereas its inhibition blocks the neurotrophic effects of BDNF on myelin-inhibited neurite outgrowth [58,60,154].

### 5.1. PACAP

**Figure 4:**
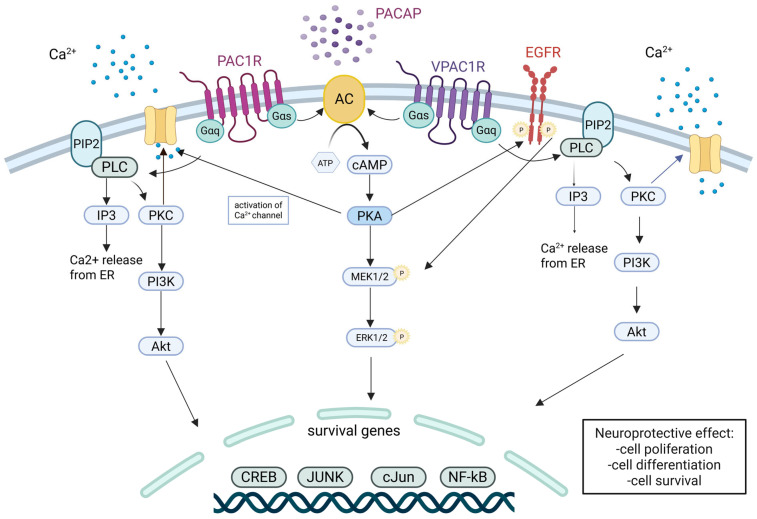
Pituitary Adenylate Cyclase-Activating Polypeptide (PACAP) receptor-mediated signaling pathways promoting neuroprotection. PACAP exerts neuroprotective effects through activation of its high-affinity receptor PAC1R (PACAP type I receptor) and low-affinity receptors: VPAC1R (vasoactive intestinal peptide receptor 1) and VPAC2R (vasoactive intestinal peptide receptor 2), which are coupled with G-proteins (Gαs and Gαq). Upon PACAP binding, these receptors activate adenylyl cyclase (AC), leading to cyclic adenosine monophosphate (cAMP) production and protein kinase A (PKA) activation. PKA phosphorylates downstream targets including MEK1/2 (mitogen-activated protein kinase kinase 1/2) and ERK1/2 (extracellular signal-regulated kinase 1/2), which translocate to the nucleus to regulate transcription of survival genes such as CREB (cAMP- response element-binding protein), JUN, cJUN (jun proto-oncogene), and NF-κB (nuclear factor kappa-light-chain-enhancer of activated B cells). In parallel, PACAP receptors also engage the PLC/IP_3_/PKC signaling cascade via Gαq. This includes phospholipase C (PLC) activation, generation of inositol 1,4,5-trisphosphate (IP3), and stimulation of protein kinase C (PKC), leading to calcium (Ca^2+^) release from the endoplasmic reticulum (ER). This calcium signaling supports activation of the phosphoinositide 3-kinase/protein kinase B (PI3K/Akt) pathway, which is crucial for cell survival and growth. Additionally, epidermal growth factor receptor (EGFR) transactivation may amplify PLC-dependent signaling. Collectively, these pathways contribute to PACAP’s neuroprotective functions, including cell proliferation, differentiation, and resistance to apoptosis. Created in BioRender. Tomczak, J. (2025) https://BioRender.com/mt2mtgo.

Pituitary adenylate cyclase-activating polypeptide (PACAP) is a highly conserved neuropeptide that indirectly stimulates adenylyl cyclase through activation of Gs-coupled receptors (Gs-GPCRs). It is widely expressed throughout the central nervous system, including key regions such as the hippocampus, cortex, and hypothalamus [155]. Among the numerous Gs-GPCR agonists capable of elevating intracellular cAMP levels, PACAP is distinguished by its exceptional potency and broad spectrum of its neuroprotective and neuroregenerative effects. PACAP enhances neuronal survival and axonal regeneration by activating AC and subsequently engaging the PKA/CREB transcriptional axis. In addition, it modulates several key cellular processes relevant to nervous system injury and neurodegeneration, including neuroinflammation, calcium signaling, and mitochondrial function [156,157]. Beyond the PKA pathway, PACAP-initiated cAMP signaling also activates additional cascades such as MAPK and phospholipase C (PLC) pathways, which are critical for cell survival, differentiation, and axonal growth [158,159]. These effects are mediated via three G-protein-coupled receptors— pituitary adenylate cyclase-activating polypeptide type I receptor (PAC1), vasoactive intestinal peptide receptor 1 (VPAC1), and vasoactive intestinal peptide receptor 2 (VPAC2)—each with distinct signaling properties. While PAC1 and VPAC1 receptors are coupled with AC, leading to cAMP production, they also engage PLC to stimulate Ca^2+^ mobilization and PKC activation, influencing cellular responses such as growth and survival [160,161,162,163]. The activation of these pathways by PACAP is tissue- and development-stage-specific, indicating the complexity of its role in both normal physiology and neuroregenerative processes [164,165] (Figure 4). Collectively, these attributes make PACAP not only a representative but also an exceptionally well-characterized example of indirect AC activation with high translational relevance for neuroregenerative strategies.

In the peripheral nervous system, PACAP plays an essential role in promoting axonal regeneration following injury. When peripheral nerves are damaged, PACAP levels increase in the distal nerve stump and DRG neurons, which facilitates axonal outgrowth and regeneration [166,167]. This regenerative effect is mediated by PACAP’s ability to stimulate Schwann cells, which are vital for the repair and remyelination of damaged axons. PACAP enhances the expression of myelin-related genes in Schwann cells and regulates the inflammatory response in the injured nerve, contributing to both remyelination and the resolution of inflammation [168,169]. Single-cell RNA sequencing of DRG neurons revealed Adcyap1 as a protective factor linking pain and nerve regeneration after injury. Intrathecal administration of PACAP38, the protein encoded by Adcyap1, mitigated pain and facilitated axonal regeneration in a rat model of spared nerve crush [167]. These actions highlight PACAP’s essential role in post-injury recovery and nerve regeneration. Moreover, PACAP’s ability to modulate inflammation in the injured peripheral nerve further underscores its importance in promoting a regenerative environment, as it inhibits the release of pro-inflammatory cytokines and enhances the expression of anti-inflammatory cytokines, which is vital for tissue repair and regeneration [169,170].

In the central nervous system, PACAP exerts robust neuroprotective effects that are critical in a variety of neurological conditions, including stroke, neurodegenerative diseases, and traumatic brain injuries. The neuroprotective actions of PACAP are largely attributed to its activation of PAC1 receptors, which initiate a series of intracellular events aimed at preserving neuronal integrity. These include the regulation of ionic homeostasis, reduction in excitotoxicity, and inhibition of oxidative stress, all of which help to prevent neuronal death in the face of injury [159,171]. For instance, PACAP has been shown to protect neurons from ischemic damage in models of stroke by modulating NMDA receptor subunits and reducing calcium overload, a major cause of cell death following ischemic events [172]. In Parkinson’s disease, PACAP plays a neuroprotective role by preventing the degeneration of dopaminergic neurons and enhancing their survival in both in vitro and in vivo models [157,173]. PACAP has also been implicated in modulating autophagy in PD, reducing autophagic activity, and protecting neurons from the toxic effects of accumulated proteins, a hallmark of PD pathology [174,175]. These findings support PACAP’s potential as a therapeutic agent in treating neurodegenerative diseases, where neuronal death and dysfunction are prevalent. PACAP’s neuroprotective effects extend to other neurodegenerative conditions such as Alzheimer’s disease and Huntington’s disease, where it has been shown to mitigate cognitive decline and protect synaptic function [157,176]. In AD models, PACAP helps prevent the neurotoxic effects of amyloid-beta plaques, which contribute to neuronal dysfunction and loss. Through its action on the PAC1 receptor, PACAP promotes the release of neurotrophic factors like BDNF, which is essential for synaptic plasticity and cognitive function, further supporting its therapeutic potential in these diseases [177]. In addition to its neuroprotective properties, PACAP is involved in modulating inflammation and apoptosis, both of which play significant roles in the progression of neurodegenerative diseases. By reducing neuroinflammation and inhibiting apoptotic pathways, PACAP helps maintain neuronal survival and function in chronic diseases [178,179].

Despite its promising therapeutic effects, the clinical application of PACAP is hindered by challenges, particularly its rapid in vivo degradation and the peripheral side effects associated with VPAC1 and VPAC2 receptor activation, which can lead to unwanted vasodilation and increased heart rate [179]. To address these issues, researchers have developed PACAP analogs with improved stability and selectivity for the PAC1 receptor. Modified peptides such as [Ala(7)]PACAP27 and [Hyp(2)]PACAP27 have shown increased resistance to degradation and enhanced neuroprotective effects, particularly in models of PD and ischemic stroke [180]. Another challenge for PACAP-based therapies is its limited ability to cross the blood–brain barrier. To overcome this, alternative delivery strategies such as intranasal administration and nanoparticle-based drug delivery systems are being explored, aiming to increase PACAP concentration in the brain while minimizing systemic exposure [177]. Such approaches could significantly enhance PACAP’s clinical viability, allowing more effective treatment of conditions such as stroke, AD, PD, and multiple sclerosis [181]. Moreover, recent studies have highlighted PACAP’s role in neurodevelopment. PACAP promotes neurogenesis in the adult brain, particularly in the hippocampus, which is critical for learning and memory [182]. Through its action on PAC1 receptors, PACAP enhances the survival of newly generated neurons, supporting cognitive function and recovery following brain injury [183,184]. Additionally, PACAP is implicated in the regulation of synaptic plasticity, essential for learning, memory, and cognitive function, suggesting that it supports not only neuronal protection but also the generation of new neurons [185,186]. In the context of traumatic injuries, PACAP plays a crucial role in tissue repair and regeneration, particularly in spinal cord and traumatic brain injury models [187,188]. Following injury, PACAP levels rise in the damaged tissue, where they promote neuronal survival and axonal regeneration, facilitating functional recovery. The peptide’s ability to modulate inflammation and promote tissue repair further underscores its potential as a therapeutic target for both traumatic and ischemic brain injuries [156,188,189,190].

### 5.2. AC Inhibitors

Adenylyl cyclase inhibitors have recently gained attention as potential therapeutic agents for neurodegenerative diseases due to their ability to modulate intracellular signaling pathways that regulate neurogenesis, neuronal survival, and neural repair. Inhibition of AC has been shown to have significant effects on the CNS, particularly in promoting neuroregeneration [191] (Table 2). Recent studies have demonstrated that AC inhibition can enhance neural stem cell (NSC) proliferation and neural progenitor cell (NCP) activity, particularly in regions involved in neurogenesis such as the subventricular zone (SVZ). In a mouse model of Alzheimer’s disease, the administration of 2′,5′-Dideoxyadenosine, an AC inhibitor, led to a significant increase in NSC and NCP populations, which correlated with improvements in age-related cognitive and behavioral deficits. These improvements included enhanced exploratory behavior and better performance in conditioned reflex tests, suggesting that AC inhibition could restore at least some aspects of neurogenesis that are diminished in neurodegenerative conditions. This increase in neurogenic capacity was accompanied by enhanced neuroglial support, as oligodendrocytes and microglia were stimulated to secrete neurotrophic growth factors, further promoting brain repair [119]. The benefits of AC inhibition extend beyond Alzheimer’s disease. In a model of ethanol-induced neurodegeneration, chronic ethanol exposure caused significant neuropsychiatric dysfunction, including neuronal degeneration, increased motor activity, and cognitive impairments. Histological analyses revealed extensive damage in the brain, including edema, perivascular inflammation, and excitotoxicity. Administration of AC inhibitors led to marked neuroprotection, with a reduction in neuronal degeneration and restoration of NSC and NCP proliferation. This functional recovery was attributed to the modulation of cAMP signaling, which enhanced neuroregenerative processes and supported glial cell function. AC inhibition restored the balance between NSC proliferation and NCP activity, a crucial factor for brain regeneration [192].

The potential of AC inhibitors in addressing synaptic dysfunction and excitotoxicity stems largely from studies of the calcium-sensitive isoform AC1. Under physiological conditions, AC1 is activated by Ca^2+^/calmodulin and supports synaptic strength and memory formation. However, when NMDA receptors are overstimulated, excessive AC1 activity can exacerbate calcium overload and drive excitotoxic neuronal death [193]. Genetic deletion or pharmacological inhibition of AC1 has been shown to protect neurons from glutamate- and NMDA-induced toxicity: cortical cultures from AC1 knockout mice sustain less excitotoxic death, and in vivo NMDA-lesion models produce smaller cortical injuries when AC1 is absent. Moreover, AC1 knockout animals display blunted inflammatory responses, further underscoring AC1′s central role in maladaptive plasticity [193,194,195]. Because excitotoxicity—driven by excessive glutamate and dysregulated calcium—is a final common pathway in many neurodegenerative disorders, targeting AC1 offers a promising complement to existing NMDA-modulating drugs such as memantine, whose benefits remain modest [196]. Moreover, research suggests that AC inhibitors can modulate the complex cAMP/PKA signaling pathway, which regulates various aspects of neurogenesis and glial cell activity. In physiological conditions, cAMP signaling promotes NSC proliferation while inhibiting NCP mitotic activity. However, in pathological states such as ethanol-induced neurodegeneration, this balance is disrupted, impairing neurogenesis and the brain’s ability to repair itself. Inhibition of AC in these models restores the proper function of NSCs and NCPs, promoting neurogenesis and enhancing glial support mechanisms [192]. In addition to their neuroregenerative properties, AC inhibitors have shown promise in the treatment of chronic pain. AC1 has been identified as a key player in neuropathic pain, with studies demonstrating that selective inhibition of AC1 can reduce pain-related cortical potentiation and mechanical hypersensitivity in animal models of chronic pain. Unlike conventional pain treatments, which often have significant side effects, AC1 inhibitors do not impair cognitive or motor functions, making them a promising non-opioid alternative for pain management [197,198]. Screening efforts have identified novel AC1-selective inhibitor scaffolds with potential for further development [199]. In vitro cellular models have been developed to assess AC1 inhibitors’ effects on pain-related signaling pathways [200]. Notably, the selective AC1 inhibitor ST034307 has shown efficacy in relieving various types of pain in mouse models without causing analgesic tolerance after chronic dosing [201].

**Table 2:** Overview of experimental models investigating the effects of adenylyl cyclase inhibition in neuroregeneration and neuroprotection.

Experimental Model	Adenylyl Cyclase Inhibitor	Primary Outcome	References
Cell culture: rat cerebellar granule neurons	tmAC inhibitor: ddAdo sAC inhibitors: KH7 or OH-E	Pharmacological inhibition of tmACs does not interfere with BDNF-induced neurite outgrowth; sAC inhibitors block BDNF-induced neurite outgrowth in inhibitory environments	[60]
Mouse model of Alzheimer’s disease	2′,5′-Dideoxyadenosine	↑ neural-stem/neural-progenitor proliferation in the SVZ, ↑ neurotrophic-factor release, improved exploratory and conditioned-reflex performance	[119]
Mouse model of ethanol-induced neurodegeneration	2′,5′-Dideoxyadenosine	Neuroprotection: ↓ neuronal degeneration, restoration of NSC/NPC balance, improved cognition and motor activity	[202]
Mouse model of neuropathic pain	NB001	Reversal of mechanical allodynia without cognitive or motor side-effects	[203,204]
In vitro cortical cultures + in vivo NMDA cortical-lesion mouse	AC1 genetic deletion (or AC1 inhibition)	↓ glutamate- and NMDA-induced excitotoxic neuronal death	[193]
Mouse models of chronic neuropathic and inflammatory pain	NB001	Effective against chronic pain without noticeable side effects	[197]
MPTP-induced mouse model of Parkinson’s disease	NB001	Reduced chronic pain and anxiety-related behaviors without affecting motor function	[204]
In vitro: HEK293 cells expressing AC1; In vivo: mouse model of inflammatory pain	ST034307	Selective AC1 inhibition suppressed cAMP production and reduced inflammatory pain responses	[205]
Chronic inflammatory- and neuropathic-pain models (mouse)	ST034307	Dose-dependent analgesia without tolerance on chronic dosing	[201]

## 6. Conclusions and Future Perspectives

Adenylyl cyclases are critical regulators of cAMP signaling, which modulates numerous processes in the nervous system. Extensive evidence from experimental models demonstrates that AC-generated cAMP promotes transcription of pro-regenerative genes, cytoskeletal remodeling and neuronal-glial interactions that are essential for functional recovery after neural injury [58,82]. Among the ten mammalian AC isoforms, AC1, AC8, AC5, and sAC are particularly relevant to neuroregeneration due to their distinct regulatory mechanisms and distribution. AC1 and AC8, both calcium/calmodulin-sensitive, couple neuronal activity to cAMP production and contribute to synaptic plasticity, axon outgrowth, and cognitive function, though AC1 also plays a maladaptive role in chronic pain [206,207]. AC5, expressed mainly in the basal ganglia and striatum, is involved in dopaminergic signaling and has been implicated in motor control and Parkinson’s disease pathophysiology [208,209]. In contrast, sAC, which responds to intracellular bicarbonate and calcium, acts as a metabolic sensor and promotes axonal regeneration in inhibitory CNS environments. It supports neurite outgrowth by integrating ionic signals with gene transcription in response to factors like BDNF [22,60]. From a therapeutic perspective, both activation and inhibition of ACs have demonstrated clinical potential, depending on the isoform and type of disease [210]. Pharmacological activation using agents like forskolin, PACAP, and PDE4 inhibitors has enhanced axonal regrowth and neuroprotection in models of spinal cord injury, Parkinson’s disease, and multiple sclerosis [150,173,211]. Conversely, selective inhibition of AC1 using compounds such as NB001 or ST034307 has effectively reduced maladaptive synaptic potentiation and neuropathic pain without impairing normal synaptic function [197,205]. Despite these advances, several challenges impede clinical translation, including the non-selective nature of existing modulators, the blood–brain barrier’s limited permeability, and compensatory mechanisms among AC isoforms. Future efforts should focus on the development of isoform-selective AC modulators with favorable pharmacokinetic properties and central nervous system penetrance [191,210]. Combination therapies integrating AC-targeted agents with neurotrophic factors or anti-inflammatory compounds may further enhance their therapeutic potential, particularly in chronic or multifactorial conditions. Innovative delivery systems, such as intranasal administration and nanoparticle-based carriers, could significantly improve drug access to the brain and spinal cord [212,213,214].

Recent studies have utilized advanced molecular profiling techniques, such as spatial and single-cell transcriptomics, to characterize cellular responses during injury and regeneration. These approaches provide high-resolution insights into the heterogeneity and dynamics of cell populations within the nervous system [215,216,217]. It is anticipated that the integration of single-cell transcriptomics with spatial proteomics could be instrumental in mapping the cell-type- and context-specific expression patterns of AC isoforms across different stages of neural injury and repair. This knowledge will be essential for the rational development of isoform-specific and personalized therapeutic strategies targeting ACs in neuroregenerative medicine. Moreover, the roles of ACs extend beyond neurons to encompass non-neuronal cells such as Schwann cells, astrocytes, and microglia, which are critical mediators of inflammation, trophic support, and tissue remodeling. In Schwann cells, cAMP levels, regulated by AC activity, are tightly controlled during demyelination and remyelination processes. After peripheral nerve injury, cAMP levels decrease significantly, correlating with reduced AC activity, and only begin to increase during remyelination, highlighting the importance of ACs in peripheral nerve regeneration [81]. In astrocytes, ACs play a pivotal role in modulating inflammatory responses and neuroprotection. For instance, activation of ACs in astrocytes leads to increased cAMP production, influencing the release of neurotrophic factors and cytokines, thereby affecting neuronal survival and inflammation [218,219]. Microglia, the resident immune cells of the central nervous system, also express ACs that modulate their activation states. Elevated cAMP levels in microglia, resulting from AC activation, have been associated with a shift towards an anti-inflammatory phenotype, reducing the production of pro-inflammatory cytokines and promoting tissue repair [220,221]. These findings suggest that AC signaling in glial cells can significantly influence regenerative outcomes. Understanding how ACs modulate the function of these supporting cells could open new avenues for therapeutic intervention that surpass neuron-centric strategies.

In summary, adenylyl cyclases represent a versatile and promising class of targets in regenerative neuroscience. Their isoform-specific roles in regulating neuroplasticity, injury response, and pathological remodeling offer numerous opportunities for therapeutic exploitation. Continued investigation of the molecular specificity, physiological relevance, and pharmacological modulation of AC isoforms will be key to advancing treatments for neurological injuries and neurodegenerative diseases.

## Figures and Tables

**Table 1:** Overview of adenylyl cyclase isoforms in the nervous system.

Adenylyl Cyclase Group		Endogenous Activators	Endogenous Inhibitors	Localization in the Nervous System	Function in the Nervous System	References
ADCY1	I	Ca^2+^/CaM, PKC, Gsα	Gαi, Gαz, Gαo, Gβγ	Neurons in cortex, hippocampus, cerebellum; moderate in glial cells	Synaptic plasticity, memory formation, nociception modulation	[12,13]
ADCY2	II	PKC, Gβγ, Gsα		Neurons and astrocytes in cortex, hippocampus, thalamus	Memory encoding, neuronal maturation	[13,14]
ADCY3	I	Ca^2+^/CaM, Gsα, PKCα	CaMKII, Gβγ	Olfactory neurons, DRG neurons, primary cilia; neurons and glia in brain	Olfactory transduction, neurodevelopment, learning and memory	[13,15]
ADCY4	II	PKC, Gβγ, Gsα	PKCα	Low brain-wide; vascular endothelial cells, hippocampus	Possible synaptic plasticity role, limited olfactory role	[13,16]
ADCY5	III	PKC, Gβγ, Gsα	PKA, Ca^2+^, Gαi, Gαz	Striatum, olfactory cortex, cortex; GABAergic neurons	Motor learning, mood regulation, striatal function	[17]
ADCY6	III	Gβγ, Gsα	PKA, PKC Ca^2+^, Gαi, Gαz	Limbic system (amygdala, hippocampus), striatum; neurons, glia	Myelination, β-adrenergic signaling, axon maintenance	[13,18]
ADCY7	II	PKCα, Gsα, Gβγ		Thalamus, hypothalamus, hippocampus; microglia, neurons	GABAergic signaling modulation, stress and depression responses	[13,19]
ADCY8	I	Ca^2+^/CaM, PKC	Gβγ	Hippocampus, hypothalamus, olfactory bulb, cerebellum; neurons, OPCs	Axon guidance, synaptic plasticity, stress reactivity	[12,13,20]
ADCY9	IV	Gsα	Calcineurin, PKC	Widespread in brain; neurons and glia	Potential role in spatial memory, cognitive processes	[13,21]
ADCY10	sAC	HCO^3−^, Ca^2+^		Astrocytes, choroid plexus, cortex neurons and hippocampus	Axon growth, astrocyte metabolism, CSF regulation	[13,22,23]
